# Gaussian random fields as an abstract representation of patient metadata for multimodal medical image segmentation

**DOI:** 10.1038/s41598-025-03393-x

**Published:** 2025-05-29

**Authors:** Bill Cassidy, Christian McBride, Connah Kendrick, Neil D. Reeves, Joseph M. Pappachan, Shaghayegh Raad, Moi Hoon Yap

**Affiliations:** 1 Department of Computing and Mathematics, Manchester Metropolitan University, M13 9WL Manchester, UK; 2 Medical School, Lancaster University, Lancaster, LA1 4YW UK; 3 Lancashire Teaching Hospitals NHS Foundation Trust, PR2 9HT Preston, UK

**Keywords:** Computer science, Diabetes

## Abstract

Growing rates of chronic wound occurrence, especially in patients with diabetes, has become a recent concerning trend. Chronic wounds are difficult and costly to treat, and have become a serious burden on health care systems worldwide. Innovative deep learning methods for the detection and monitoring of such wounds have the potential to reduce the impact to patients and clinicians. We present a novel multimodal segmentation method which allows for the introduction of patient metadata into the training workflow whereby the patient data are expressed as Gaussian random fields. Our results indicate that the proposed method improved performance when utilising multiple models, each trained on different metadata categories. Using the Diabetic Foot Ulcer Challenge 2022 test set, when compared to the baseline results (intersection over union = 0.4670, Dice similarity coefficient = 0.5908) we demonstrate improvements of +0.0220 and +0.0229 for intersection over union and Dice similarity coefficient respectively. This paper presents the first study to focus on integrating patient data into a chronic wound segmentation workflow. Our results show significant performance gains when training individual models using specific metadata categories, followed by average merging of prediction masks using distance transforms. All source code for this study is available at: https://github.com/mmu-dermatology-research/multimodal-grf

## Introduction

Chronic wounds are a serious condition that can expose patients to infection and potentially increased mortality risk^1^. The global diabetes epidemic is an important factor in the case of chronic wounds, as patients with diabetes are both at increased risk of developing such wounds and are likely to experience significantly impaired healing rates^2^.

Patients who have been diagnosed with diabetic foot ulcers (DFU) have been shown to have significantly greater mortality risk when compared to those without^3^. Such patients are also more likely to suffer from additional comorbidities such as cardiovascular disease, peripheral arterial disease, retinopathy, and neuropathy^4–8^.

Arterial leg ulcers (ALU), DFU, and venous leg ulcers (VLU) can lead to impaired quality of life^9,10^. Occurrence of such wounds is associated with an incidence increase of amputation and subsequent mortality risk. These factors are particularly prevalent in older patients, and those suffering from anemia and peripheral artery disease^9,11,12^. Prevalence of chronic wounds is linked to increased occurrence of emotional and physical burdens on patients^13,14^. Depression is also commonly associated with patients with chronic wounds^15,16^.

In an effort to meet these increased demands on clinics and hospitals, there has been a growing research interest concerning non-contact automated deep learning detection and monitoring of chronic wounds^17–19^. The utilisation of deep learning methods to provide a means of early detection and remote wound monitoring could be a gateway to help reduce risks to patients who are vulnerable, and to ease the burdens that clinics and hospitals are currently experiencing^20^. Low cost consumer mobile devices can be used to bring such technologies to patients living in poorer regions, where access to expert healthcare services may be limited. Such advances could also be used to promote patient engagement with their health, a facet of patient care that has been shown to be an effective treatment strategy^21^. With these recent advances in mind, the research objective of the present paper is to explore a novel method of integrating patient metadata into a segmentation workflow as a means of enhancing chronic wound segmentation performance. Currently, there are no chronic wound segmentation methods that utilise patient metadata, which highlights a gap in the current research.

## Related work

Recent years have seen researchers focusing on utilising patient metadata in convolutional neural network (CNN) training workflows in numerous medical imaging domains. In this section, we explore the most prominent of the most relevant studies.

### Metadata

Lemay et al.^22^ adapted a feature-wise linear modulation conditioning method for medical image segmentation enabling the integration of metadata into U-Net spinal cord tumour segmentation models. The metadata is used to modulate the segmentation process using low-cost affine transformations which are then applied to feature maps during training which can be used in any CNN architecture. They found that the application of a single metadata item (tumour type) as an additional input into the segmentation network provided a 5.1% boost to performance.

Anisuzzaman et al.^23^ used wound location data to improve performance of a multi-class wound classification model using two publicly available chronic wound datasets (AZH and Medetec) and a private wound location metadata dataset. For the corresponding categorical body map metadata, each wound location was converted to an integer value and encoded using one-hot encoding. Two main models were trained, a CNN for the wound images, and a second for the wound location metadata. The CNN utilised transfer learning with VGG-16, VGG-19, ResNet-50 and IncpetionV3 sub-models together with an AlexNet. The wound location model utilised a Multi-Layer Perceptron (MLP) and a Long Short-Term Memory (LSTM) model. The output of the pretrained CNNs was concatenated with the outputs of AlexNet, the MLP, and the LSTM to form the final predictions. Their experiments demonstrated an improvement in classification performance from 72.95% to 97.12% when body map metadata features were introduced into the training workflow. They also completed experiments using the one-hot vector as a direct input into the CNN dense layer. However, this resulted in inferior results when compared to utilising the MLP or the LSTM.

Patel et al.^24^ would later build on the prior work completed by^23^. They proposed an improved multi-class multi-modal network architecture utilising parallel spatial and channel-wise squeeze and excitation, axial attention, and an adaptive gated MLP. These modifications allow the network to capture global contextual information by focusing on channel interdependencies, learning patterns across different input channels. Spatial information is also maintained by focusing on the spatial interdependencies of individual channels. Using the AZH and Medetec datasets they achieved an accuracy of 73.98-100% for classification using assisting location metadata. However, as per the previous study conducted by^23^, the dataset used was relatively small, with just 930 AZH and 538 Medetec images.

Gu et al.^25^ proposed a multimodal architecture capable of simultaneous segmentation and diagnosis of tumours using images and patient metadata. Their architecture comprised of three parts: an image encoder, a text encoder, and a decoder utilising an interlaced sparse self-attention mechanism. Text embedding features are fused at several network layers with the image data between the two encoders. The text preprocessing block embeds the metadata using a language model with bidirectional encoder representations from transforms (BERT). Each word is converted into a two-dimensional vector, with a $2\times$ upsample applied using deconvolution so that the size matches that of the input images. They reported significant improvements for basal cell carcinoma segmentation on two private datasets: +14.3% IoU and +8.5% DSC on the ZJU2 dataset, and +7.1% IoU on the LIZHU1 dataset. They also demonstrated state of the art performance on the GlaS dataset for gland segmentation in colon histology images (DSC +0.0018, IoU +0.0027). A major limitation of this work, however, is the limited size of the datasets used, with each dataset comprising fewer than 200 images.

Research of this nature demonstrates the potential of the inclusion of metadata in the development of multimodal CNNs. However, as indicated by previous research, there is a severe lack of publicly available multimodal datasets, an issue which is even more apparent in the case of chronic wounds.

### Random fields

Random fields are a generalised form of a stochastic field where randomness is determined as a function of spatial variables. Essentially, they encompass a random variation of measurable properties^26^. Gaussian random fields (GRF) are a type of random field that provide a statistical tool to describe naturally occurring structures exhibiting spatially varying uncertainties^27^. They represent a description of uncertainties that can exert critical impacts on the overall performance of physical processes found throughout nature^28^. GRFs are used to model uncertainties such as material properties, measurement errors, and distributions of attributes associated with living organisms. Treating such uncertainties as random fields or random variables, statistical analyses can be utilised more consistently^29,30^. Practical applications of GRFs include modelling of landscapes in ecology and generation of cloud features in geoscience^31^. Variants of GRFs, known as Euclidean bosonic massless free fields, are used for modelling random surfaces and functions in quantum field theory^32^.

GRFs have previously been used in active learning and semi-supervised training processes. Zhu et al.^33^ proposed a GRF text and digit classification model defined with respect to a weighted graph which represented labelled and unlabelled data. Their framework exploited unlabelled data structures to enhance classification accuracy. However, these experiments predate more modern deep learning methods.

In more recent works, Yang et al.^34^ conducted classification experiments on synthetic aperture radar images and improved performance using Markov random fields (MRF). MRF is a random field variant of GRF that introduces a Markov property which models a prediction based only on the current state and not prior or later states. This work proposed the generation of a probability field which describes regional relationships. They derived the energy function using the intensity field and the probability field, allowing for superior initialisation of the MRF.

GRFs have been used in multiple scientific disciplines, however, to the best of our knowledge they have not been used to represent metadata in multimodal deep learning experiments.

## Method

In this section we detail the training, validation, and testing workflow, the proposed method for generating GRFs using patient metadata, and corresponding metrics used to assess our multimodal chronic wound segmentation experiments.

### Patient metadata

Our experiments utilise the following patient metadata categories: (1) patient date of birth (DOB); (2) patient gender (male or female); (3) Health and Disability Decile (HDD). HDD values were obtained from the English indices of deprivation 2019 public records^35^ using patient post codes as reference. All patient metadata are present for all associated chronic wound images, with no missing instances.

### Data normalisation

**Fig. 1:**
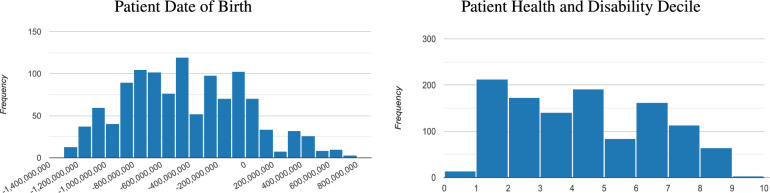
Histogram analysis of the patient date of birth and patient health and disability decile metadata present in the multimodal dataset used in our experiments. Non-normal distribution is demonstrated in both types of data. Note that patient date of birth values are represented as timestamps.

We completed a histogram analysis of the DOB and HDD patient metadata to determine how the data should be represented in our deep learning experiments. This analysis showed that the DOB and HDD patient metadata categories did not exhibit normal distributions (see Fig. 1). Therefore, the patient DOB and HDD metadata used in our experiments were normalised using min max scaling as defined in Eq. 1.

1 $$\begin{aligned} X^{'} = \frac{X - X_{min}}{X_{max}-X_{min}} \end{aligned}$$

where *X* is the data point and $X_{min}$ and $X_{max}$ are the minimum and maximum values present in the group respectively.

Patient gender was excluded from this analysis as there were only two possible values from the data provided, which we encoded as 0 (female) and 1 (male).

### Gaussian random field generation

In this section we specify how GRF images were generated to encode patient metadata as spatially structured representations, enabling integration into deep learning-based multimodal chronic wound segmentation. This approach transforms numerical metadata into synthetic images that capture spatial correlations, ensuring compatibility with CNN architectures. The GRFs are constructed using a spectral synthesis approach, leveraging the properties of the Fourier transform to generate stochastic fields with controlled smoothness and variability^36^.

Mathematically, a GRF is defined as a stochastic process *X*(*s*) over a spatial domain *S*, such that any finite collection of values $\{X(s_1), X(s_2), ...,X(s_n)\}$ follows a multivariate normal distribution. Formally, for any finite set of points $\{s_1, s_2, ..., s_n\}$, the joint distribution of *X*(*s*) is given by:

2 $$\begin{aligned} \left( X(s_1), X(s_2), ..., X(s_n)\right) \sim \mathcal {N}(\mu , K) \end{aligned}$$

where $\mu$ is the mean function $\mathbb {E}[X(s)]$ and *K*(*s*, *t*) is the covariance function that defines the dependency between the points in the field. The covariance function must be positive semidefinite to ensure a valid Guassian process.

The spatial structure of a GRF is characterised by its power spectrum $P_k$, which governs the correlation length and smoothness of the field. In this study, the power spectrum is dynamically determined based on the normalised patient metadata values. The power spectrum is computed as follows:

3 $$\begin{aligned} P_k = -\left| i + f \right| \end{aligned}$$

where *i* is an integer component that defines the global structure and smoothness of the GRF, and *f* is the fraction of the normalised metadata value. For binary nominal categorical variables such as gender, *f* is set to zero, ensuring that the GRFs remain distinct for each category. Lower values of $P_k$ result in highly fragmented structures, whereas higher values produce smoother GRFs.

The GRF generation process follows a spectral synthesis approach, which constructs a two-dimensional Gaussian noise field in the frequency domain using the Fast Fourier Transform (FFT). This noise field is modulated by the power spectrum $P_k$ to introduce spatial correlations, ensuring that local variations follow the prescribed smoothness constraints. The amplitude of the Fourier components is modified using the following equation:

4 $$\begin{aligned} A(k_x,k_y) = \sqrt{P_k(k_x, k_y)} \end{aligned}$$

where $k_x, k_y$ are the frequency components in the Fourier domain. This transformation ensures that low-frequency components are dominant within the field, generating large-scale spatial structures. High-frequency components contribute fine-grained details, but their influence diminishes as $P_k$ increases.

Once the spectral domain representation is obtained, the inverse FFT is applied to transform the frequency-modulated field back into the spatial domain. The result is a two-dimensional GRF, expressed as a greyscale image, where the pixel intensities correspond to the metadata-driven stochastic process.

**Fig. 2:**
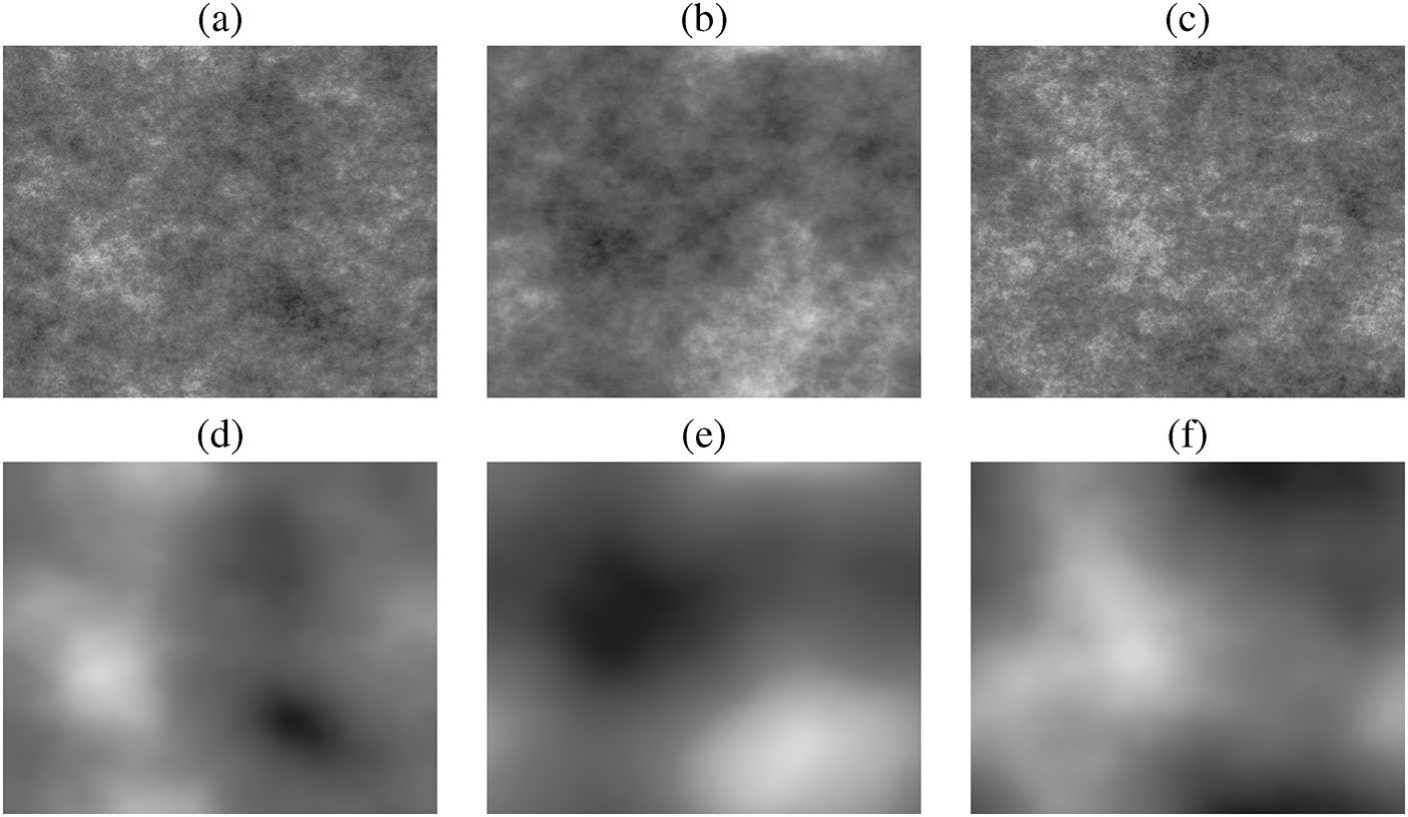
Illustration of the types of Gaussian random fields generated for use in our multimodal chronic wound segmentation experiments. Examples (**a**), (**b**), and (**c**) were generated with an *i* value of 2, and examples (**d**), (**e**), and (**f**) were generated using an *i* value of 5. The first column examples were generated using DOB, the second column examples were generated using gender, and the third column examples were generated using HDD.

All experiments used one of two types of GRF, where $i = 2$ (see Fig. 2 (a, b, and c)), or $i = 5$ (see Fig. 2 (d, e, and f)). The difference lies in their structural complexity and smoothness, where GRFs generated with $i = 2$ exhibit greater fragmentation with localised variations and more fine granularity, making them suitable for capture of highly dynamic spatial dependencies. In contrast, GRFs with $i = 5$ result in smoother and more uniform structures, which are better suited to encode broader trends and continuous variations in the metadata. The choice of *i* directly impacts the level of details within the GRF representation, with lower values producing noisier patterns and higher values resulting in more coherent spatial structures.

To ensure reproducibility and consistency across experiments, a fixed random seed is assigned for each metadata category: **DOB** (76539635), **gender** (88118546), and **HDD** (41094303). Each GRF is stored as a $640\times 480$ pixel greyscale image and merged with the RGB wound image tensors to form a four-channel input. This approach allows CNNs to extract metadata-related features without requiring direct numerical encoding, preserving an image-based representation paradigm within the segmentation pipeline. The generated GRFs act as an abstract representation of metadata, allowing the model to leverage spatial dependencies while maintaining compatibility with standard convolutional architectures.

### Metrics

To evaluate the performance of our multimodal chronic wound segmentation models we utilise a range of widely used metrics. Intersection over union (IoU) and Dice similarity coefficient (DSC) were used as the metrics for ascertaining segmentation model accuracy. DSC was selected for its representation as the harmonic mean of precision and recall, providing a more balanced evaluation of false positive and false negative prediction results. The mathematical expressions for IoU and DSC are shown in Eqs. (5) and (6) respectively.

5 $$\begin{aligned} & IoU = \frac{|X \cap Y|}{|X| + |Y|} \end{aligned}$$

6 $$\begin{aligned} & DSC = 2 * \frac{|X \cap Y|}{|X| + |Y|} \end{aligned}$$

where *X* and *Y* indicate the ground truth and predicted masks, respectively.

We also use two statistical hypothesis test metrics to provide an improved understanding of Type I and Type II errors found in deep learning segmentation algorithm results: False Positive Error (FPE) as detailed in Eq. (7), and False Negative Error (FNE) as detailed in Eq. (8).

7 $$\begin{aligned} & FPE = \frac{FP}{FP + TN} \end{aligned}$$

8 $$\begin{aligned} & FNE = \frac{FN}{FN + TP} \end{aligned}$$

where *FP* represents the total number of false positive predictions, *TN* is the total number of true negative predictions, *FN* is the total number of false negative predictions, and TP is the total number of true positive predictions.

### Chronic wound datasets

For our multimodal segmentation experiments we use a new private chronic wound dataset obtained from Lancashire Teaching Hospitals NHS Foundation Trust, UK. The use of this dataset was approved by the NHS Research Ethics Committee and the Health Research Authority (REF: SE-281). All data acquisition methods were performed in accordance with the guidelines and regulations set out by NHS Research Ethics Committee and the Health Research Authority. Written informed consent was obtained from all participating patients. This new multimodal dataset was collected between January 2023 and December 2023 during patient clinical appointments. A total of 1142 chronic wound images were captured using three digital cameras: a Kodak DX4530 (5 megapixel), a Nikon COOLPIX P100 (10.3 megapixel), and a Nikon D3300 (24.2 megapixel). Auto-focus was used during capture with no zoom or macro functions active, with an aperture setting of f/2.8 at a distance of approximately 30-40 cm from the wound surface. Natural lighting in the hospital settings was used instead of a flash. All chronic wound images were acquired by medical photographers whose specialisation is chronic wounds, all with more than 5 years professional clinical experience. Patient data was captured by clinicians who recorded the patient’s DOB, gender, and post code. Ground truth masks were generated using the HarDNet-CWS segmentation model proposed by^37^. Therefore, all training and validation experiments completed in this study are to be considered weakly supervised. The new chronic wound dataset is used in our experiments for training and validation. For testing, we use the DFUC 2022 test set which comprises 2000 DFU wound images and associated ground truth masks^38^. The DFUC 2022 test set does not have any associated metadata. A summary of the composition of characteristics of the training set is summarised in Table 1. Note that multiple wound images may have been collected for a single patient during a hospital appointment. Such cases may include images of a single wound or multiple wounds, and may include images of the same wound captured at different angles and distances.

**Table 1:** Baseline characteristics for the multimodal training dataset comprising chronic wound images with corresponding patient metadata and weakly supervised ground truth masks. Note that in two cases it was not possible to exactly identify the wound type - “arterial or venous” and “venous or pressure”. $^*$ - with fungal component.

Category	Total	Category	Total
No. of wound images	1142	No. of venous or pressure wound images	1
No. of DFU wound images	1111	No. of patients	308
No. of venous wound images	13	No. of appointments	94
No. of arterial wound images	12	No. of male patients	229
No. of pressure wound images	1	No. of female patients	79
No. of dermatoliposclerosis wound images	1	Median patient age	70
No. of bacterial infection wound images$^*$	1	Median male patient age	69
No. of ulcer on necrobiosis lipoidica wound images	1	Median female patient age	70
No. of arterial or venous wound images	1		

### Baselines

We use a selection of commonly used segmentation architectures to generate a set of baseline results to ascertain which architecture will be used in our multimodal experiments. Included in the baseline experiments is the HarDNet-CWS network architecture, which was proposed in our prior chronic wound segmentation works^37^. HarDNet-CWS is a hybrid transformer segmentation architecture that uses traditional convolutional techniques in the encoder, and a vision transformer in the decoder. This model is based on HarDNet-DFUS which was the winning entry for the DFUC 2022. No augmentation or post-processing methods were used in any of the baseline experiments. Pretrained weights were also not used. For the baseline experiments, only wound images were used for training, validation, and testing.

### Model parameters

The non-HarDNet-CWS baseline experiments were trained for 100 epochs at a batch size of 8 using the AdamW optimiser with a learning rate of 0.001 and a weight decay of 0.0001. The HarDNet-CWS baseline and subsequent experiments were trained for 60 epochs at a batch size of 5 using the AdamW optimiser with a learning rate of 0.00001, an epsilon of 0.0000001, and a weight decay of 0.01. The AdamW optimiser was selected as it enables the decoupling of weight decay from gradient updates, allowing for improved generalisation, enhanced regularisation and more stable convergence^39^. We initially experimented using the same parameters for all baseline models, however, due to significantly differing architecture designs, we found the non-HarDNet models to perform better using the parameters described here.

### Hardware and software configuration

The following hardware and software configuration was used in all our experiments: Debian GNU/Linux 10 (buster) operating system, AMD Ryzen 9 3900X 12-Core CPU, 128GB RAM, NVIDIA GeForce RTX 3090 24GB GPU. Models were trained with Pytorch 1.13.1 using Python 3.7.13.

### Training with gaussian random fields using tensor merging

Inspired by recent multi-colour space tensor merging experiments conducted by^40^, we experiment by introducing the GRF images into the training workflow by merging single channel GRFs with the RGB tensors representing the actual wound images. The tensor merging process was completed by merging the GRF single channel tensor onto the end of the RGB tensor which forms a new 4D tensor. This process is detailed in Algorithm 1 which shows a pseudo code summary of the RGB and GRF tensor merging process. Early fusion was selected to ensure that inter-modal interactions occur throughout the network during training, allowing for richer feature representations to be learnt. For experiments where GRFs were introduced into the training and validation workflow, inference on the test set used a zeroed tensor channel in place of the metadata features not present in the test set. The zeroed tensor channel was set to the same dimensions as the input images during testing.

**Algorithm 1:**
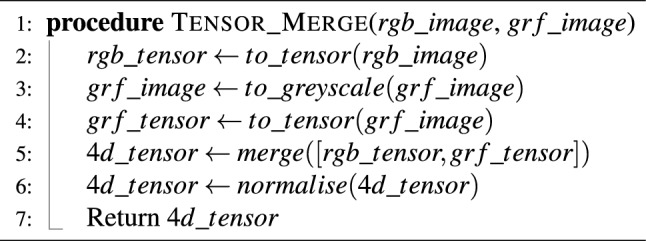
RGB+GRF tensor merging algorithm.

### Average merging of prediction masks

**Fig. 3:**
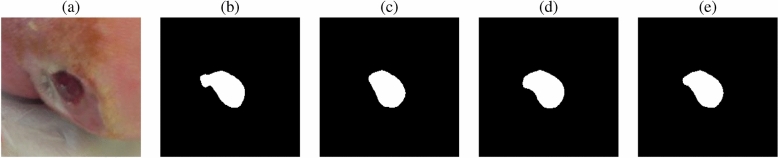
Illustration of the prediction mask average merging process completed using distance transforms: (**a**) shows the original wound image, (**b**) is a mask generated by the model trained using DOB GRFs, (**c**) is a mask generated by the model trained using gender GRFs, (**d**) is a mask generated by the model trained using HDD GRFs, and (**e**) is the average merged mask. Note that images have been cropped for illustrative purposes.

To further enhance prediction results, we complete a series of experiments whereby prediction masks are merged, using average merging, from predictions generated for the test set for the models trained using $i = 2$ and $i = 5$. Three sets of results are produced: (1) average merging of prediction results for the models trained using GRFs generated from DOB, gender, and HDD metadata where $i = 2$; (2) average merging of prediction results for the models trained using GRFs generated from DOB, gender, and HDD metadata where $i = 5$; and (3) average merging of prediction results for the models trained using GRFs generated from DOB, gender, and HDD metadata where $i = 2$ and $i = 5$. Prediction masks were averaged using the OpenCV distance transform method^41^. The distance transform calculation is shown in Eq. 9. An example of prediction mask average merging using distance transforms is shown in Fig. 3.

9 $$\begin{aligned} \begin{aligned} D_p(p) = \min _{q \in G} (d(p,q)+1(q)) \\ 1(q) = {\left\{ \begin{array}{ll} 0 & \text {if }q \in P\\ \infty & \text {otherwise}\\ \end{array}\right. } \end{aligned} \end{aligned}$$

where *P* is a set of points on grid *G* ($P \subseteq G$), and associates to each grid location $q \in G$ the distance to the nearest point $p \in P$.

## Results

In this section we report on the results of inference for the baseline model, which was trained using only wound images, and the models trained using wound images with the patient metadata expressed as GRFs.

### Baseline results

The training, validation, and test results for the baseline models are summarised in Table 2. The HarDNet-CWS model was shown to be the highest performing model in these experiments, and was therefore selected as the basis for all subsequent experiments.

**Table 2:** Results for the baseline models trained and validated using only wound images from the new multimodal dataset, and tested on the DFUC 2022 test set, which also comprises only wound images. All wound images are $640 \times 480$ pixels. Ep - epoch; Tr - train; V - validation; Te- test; UN - U-Net; IoU - intersection over union; DSC - Dice similarity coefficient; FPE - false positive error; FNE - false negative error. Note that no pretraining and no pre- or post-processing was used in these experiments.

Model	Ep	Tr-IoU	Tr-Loss	Tr-DSC	V-IoU	V-Loss	V-DSC	Te-IoU	Te-DSC	FPE	FNE
VGG16 UN	84	0.7866	0.1499	0.8722	0.4602	0.4939	0.6032	0.3482	0.4652	0.0625	0.4171
ResNet50 UN	60	0.8324	0.1184	0.9039	0.4745	0.4745	0.6304	0.4007	0.5213	0.0525	0.3884
EfficientNetB0 UN	64	0.8741	0.0920	0.9301	0.5344	0.3833	0.6745	0.4603	0.5835	0.0169	**0.3482**
ConvNeXt UN	40	0.5510	0.3163	0.6967	0.3964	0.5456	0.5531	0.3288	0.4519	0.0425	0.4422
HarDNet-CWS	44	0.9346	0.0972	0.9658	0.5887	0.5246	0.7018	**0.4670**	**0.5908**	**0.0169**	0.3637

### Gaussian random field results

**Fig. 4:**
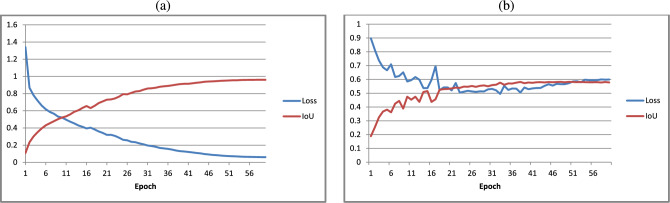
Illustration of the training (**a**) and validation (**b**) loss curves for the HarDNet-CWS model trained and validated using HDD GRFs with an *i* value of 2.

The results for the chronic wound segmentation experiments, where the patient metadata expressed as single channel greyscale GRF images was included into the training workflow, are summarised in Table 3. Train and validation loss curves for the highest performing model (HDD, $i=2$) are shown in Fig. 4.

**Table 3:** Results for the HarDNet-CWS model trained and validated using the new multimodal dataset, and tested on the DFUC 2022 test set (image size = $640 \times 480$ pixels) using GRFs. *i* - default value of GRF power spectrum integer component; IoU - intersection over union; DSC - Dice similarity coefficient; FPE - false positive error; FNE - false negative error; DOB - date of birth; HDD - health and disability decile. Note that no pretraining, augmentation, or post-processing was used in these experiments.

Metadata	*i*	Epoch	Train IoU	Train Loss	Train DSC	Val IoU	Val Loss	Val DSC	Test IoU	Test DSC	FPE	FNE
DOB	2	44	0.9323	0.1012	0.9647	0.5615	0.5539	0.6760	0.4699	0.5944	0.0169	0.3562
DOB	5	54	0.9566	0.0670	0.9778	0.5654	0.5866	0.6842	0.4652	0.5894	0.0169	0.3475
Gender	2	34	0.8810	0.1659	0.9359	0.5554	0.5277	0.6708	0.4703	0.5942	0.0169	0.3575
Gender	5	36	0.8843	0.1635	0.9376	0.5832	0.5038	0.6976	0.4641	0.5889	0.0169	0.3621
HDD	2	47	0.9454	0.0834	0.9718	0.5825	0.5657	0.6962	**0.4706**	**0.5946**	**0.0169**	**0.3424**
HDD	5	48	0.9394	0.0893	0.9685	0.5672	0.5652	0.6819	0.4626	0.5873	0.0169	0.3465

### Gaussian random field experiment predictions with average merging

The results of the GRF experiments where predictions from different models, each trained and validated on a single GRF type and combined using average merging, are summarised in Table 4.

**Table 4:** Test results for the HarDNet-CWS models with averaged prediction masks. *i* - default value of GRF power spectrum integer component; IoU - intersection over union; DSC - Dice similarity coefficient; FPE - false positive error; FNE - false negative error; DOB - date of birth; Gen - gender, HDD - health and disability decile. Note that no pretraining, augmentation, or post-processing was used in these experiments.

Metadata	*i*	IoU	DSC	FPE	FNE
DOB+Gen+HDD	2	**0.4899**	**0.6128**	**0.0199**	0.3209
DOB+Gen+HDD	5	0.4841	0.6079	0.0199	0.3192
DOB+Gen+HDD	2 & 5	0.4897	0.6122	0.0254	**0.3171**

## Discussion

The results of models trained using RGB wound images and patient metadata expressed as GRFs (see Table 3) indicate that for all models trained where $i = 2$, improvements in terms DSC were demonstrated when compared to the baseline results. The best overall performing GRF model was the HDD model ($i = 2$) which demonstrated improvements of 0.0036, 0.0038, and 0.0213 for IoU, DSC, and FNE respectively when compared to the baseline results. We also observe that in terms of FPE, the baseline and subsequent experiment results are unchanged, with a reported value of 0.0169. The IoU and DSC improvements for these experiments are marginal ($< 1\%$), whereas we would consider the improvement of FNE to be significant ($> 2\%$).

For the experiments using averaged prediction masks from all model types (DOB, gender, and HDD - see Table 4), we observe that the models trained with $i = 2$ provided the highest performance improvements. The averaged model predictions trained with $i = 2$ demonstrated improvements in terms of IoU (+0.0229) and DSC (+0.0220) when compared to the baseline results. When compared to the results for models trained on individual metadata categories (DOB, gender, and HDD - see Table 3), improvements are observed in terms of IoU (+0.0193) and DSC (+0.0182) for the $i = 2$ models, and FNE (-0.0253) for the combined $i = 2$ and $i = 5$ models. When compared to the models trained on individual metadata categories (see Table 3), these results indicate that increasing the number of patient metadata categories improves overall network performance in terms of IoU, DSC, and FNE. We suggest that these results are promising, considering that our experiments were conducted using metadata only for the training and validation sets.

When comparing the training loss and validation loss curves (see Fig. 4) the validation curve is clearly less stable than the training loss curve. We suggest that this may be due to the weakly supervised nature of the training and validation sets, whereby label noise affects the training process to varying degrees. This effect may also be exacerbated by the limited size of the training and validation sets.

When comparing our highest test results (IoU = 0.4899, DSC = 0.6128) with the winning entry for the DFUC 2022 (IoU = 0.6252, DSC = 0.7287) we observe a difference of 0.1353 for IoU and 0.1159 for DSC. These differences are significant, however, it should be taken into account that the experiments in the present paper use a substantially smaller training set with weakly supervised training and validation masks. Therefore, a direct comparison of these results does not compare like for like due to the substantial differences in experimental setups.

**Fig. 5:**
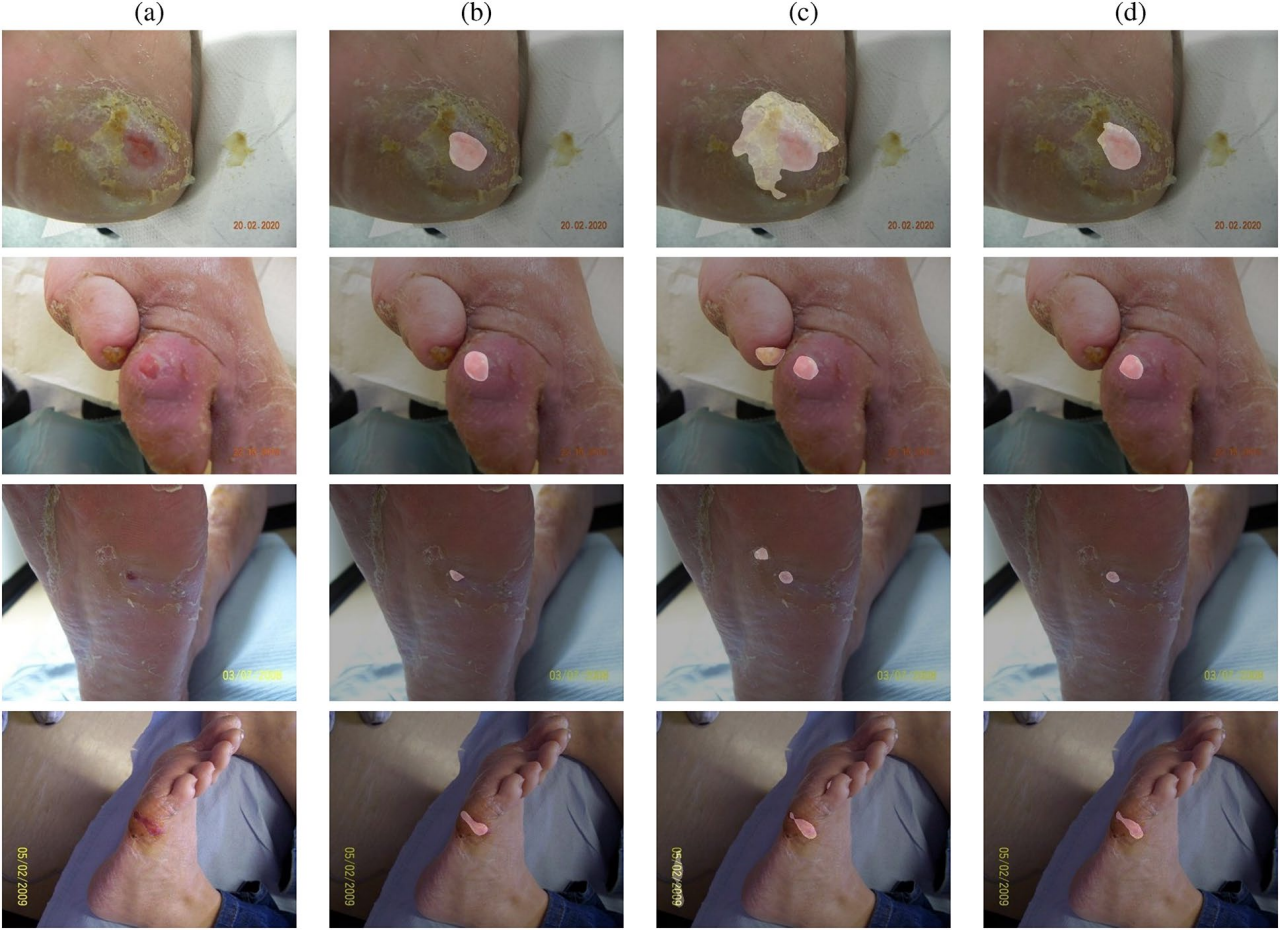
Illustration showing four examples of where the averaged merged predictions from the $i = 2$ models show improved performance over the baseline results. Column (**a**) shows the original wound image, column (**b**) shows the ground truth mask, column (**c**) shows the baseline HarDNet-CWS prediction, and column (**d**) shows the averaged merged prediction for the $i = 2$ models.

A selection of baseline and averaged merged predictions from the $i = 2$ models are shown in Fig. 5. The first row shows a case where the baseline result has incorrectly predicted parts of the dried skin surrounding the wound, whereas the averaged merged prediction is significantly closer to the ground truth mask. The second row shows a case where the wound region has been correctly predicted by both baseline and averaged merged predictions, however, the baseline has also incorrectly predicted a toenail as a wound region. The third row shows a case where the baseline and averaged merged predictions have correctly predicted the wound located on the plantar aspect, however, the baseline model has also incorrectly predicted on a region of dried skin. The fourth row shows a case where the baseline has predicted less of the wound region compared to the averaged merged predictions.

The rationale for using GRF images in our multimodal chronic wound segmentation experiments was to ensure that all input data, and therefore learnable features, remained strictly within the confines of the computer vision domain. That is, all learnable features could be derived from imaging domains, regardless of the nature of the data (image or numerical data). Generation of synthetic images to represent non-image data was previously proposed by^42^, named DeepInsight, for classification tasks. They converted feature vectors derived from text values to a feature matrix which is used to generate an image. However, their approach generated sparse pixel patterns in images that CNNs may find difficult to learn features from and may not scale well to higher resolutions. Their method was also not used in terms of multimodality. Our approach is capable of generating dense features at any resolution regardless of the complexity of the input value, and has been demonstrated in a multimodal segmentation architecture. Additionally, GRF images comprise complex texture features, of which CNNs naturally exhibit a learning bias towards^43^, which provides a further potential advantage over the method proposed by^42^.

Obtaining patient metadata is both time consuming and challenging, entailing numerous ethical hurdles. Making such datasets public also brings numerous risks regarding patient anonymity. Distribution of patient metadata as abstract representations, as proposed in the present paper, may be a way to circumvent many of the challenges associated with the distribution of patient data and may encourage other research groups to share much needed data with the research community.

Another limitation of the present study involves the limited size of the training set. The data used in our study represents both chronic wound images and associated patient metadata. With just 1142 chronic wound images, collected from 308 patients, this data is limited in size and may also exhibit bias due to the limited number of patients. However, despite these limitations, factors such as images obtained at different appointments and at different angles and orientations may to some extent circumvent the apparent biases. We also observe other imbalances in the training set, particularly in terms of male and female distribution, and to a lesser extent, median male and female patient age. Our research group is currently in the process of collecting additional multimodal data from hospitals both in the UK and internationally with a future focus on utilising such datasets in upcoming research which will build on the methods presented in the current study.

Preprocessing methods have been shown to be valuable techniques when training deep learning models in medical imaging domains^44–47^. However, we do not report on such methods in the present paper, as those methods did not provide performance benefits over the current state-of-the-art for the test set used in our experiments.

The multimodal segmentation experiments conducted in the present paper were weakly supervised. Therefore, we present these findings as preliminary research which may be used as a basis of comparison in future studies when more patient data becomes available, allowing for more extensive multimodal segmentation studies. We encourage other research groups working in multimodal medical imaging deep learning tasks to further explore the concepts presented in the present paper.

## Conclusion

In this work we propose the use of GRFs generated using patient metadata for use in a chronic wound segmentation workflow. Our results demonstrate that via an ensemble of models, each trained on different GRFs generated from different patient metadata categories, we were able to outperform the baseline experiment results (without GRFs) in terms of IoU (+0.0229) and DSC (+0.0220). These experimental results were achieved using weak supervision for the training data, in addition to the use of a training set that was significantly smaller when compared to the test set (train set = 1142; test set = 2000). Our approach allows for the introduction of patient data into multimodal CNN models with minimal adjustments to the architecture design. Additionally, we demonstrate that the test results can be improved with the use of GRFs where only the training data has associated metadata. Our findings indicate that the use of GRFs as an abstract representation of patient metadata is a viable option in deep learning training workflows for segmentation, with potential utility in classification and localisation tasks.

## Data Availability

The multimodal training and validation sets used in the present study are private and not yet publicly available. For testing, we used the publicly available DFUC 2024 segmentation test set, which is available upon request by emailing Prof. Moi Hoon Yap: m.yap@mmu.ac.uk
